# Acute kidney injury severity in ICU patients: Developing and evaluating a data-driven analysis of clinical covariates using machine learning

**DOI:** 10.1371/journal.pone.0352314

**Published:** 2026-07-16

**Authors:** Mohammad Fathi, Nader Markazi Moghaddam, Mahdis Fathi, Mohammadreza Hajiesmaeili, Navid Nooraei, Nasser Malekpour Alamdari, Sanaz Zargar Balaye Jame, Hamed Markazi Moghadam

**Affiliations:** 1 Critical Care Quality Improvement Research Center, Shahid Modarres Hospital, Shahid Beheshti University of Medical Sciences, Tehran, Iran; 2 Department of Health Management and Economics, Faculty of Medicine, Aja University of Medical Sciences, Tehran, Iran; 3 School of Pharmacy, Shahid Beheshti University of Medical Sciences, Tehran, Iran; 4 Critical Care Quality Improvement Research Center, Loghman Hakim Hospital, Shahid Beheshti University of Medical Sciences, Tehran, Iran; 5 GBD Collaborator Network, Global Labor Organization (GLO), Faculty of Economics and Management, Leibniz University Hannover, Hannover, Germany; Istituto Di Ricerche Farmacologiche Mario Negri, ITALY

## Abstract

**Background:**

Acute kidney injury remains a major cause of morbidity and mortality in critically ill patients. Existing classification systems rely on serum creatinine and urine output thresholds and do not fully capture the complex, multifactorial nature of acute kidney injury severity in the ICU.

**Objective:**

To develop and validate a machine learning model using high-dimensional clinical data to identify covariates associated with the severity of acute kidney injury and enhance early risk stratification.

**Methods:**

We performed a secondary analysis of 2,281 patients from the ICU in the MIMIC-IV database. Acute kidney injury severity was staged (0–3) based on serum creatinine criteria. After rigorous data cleaning, imputation, and feature selection, an XGBoost model was trained. Model performance was assessed, and gain-based metrics were used to identify the most important predictors of acute kidney injury severity.

**Results:**

The XGBoost model demonstrated favorable discriminative performance, with an overall accuracy of 77.6% and a mean absolute error of 0.241. It achieved high precision and recall for stage 0 (precision = 0.917) and stage 3 (recall = 0.837), but showed decreased performance for stage 2. The top 10 covariates associated with increasing acute kidney injury severity were chronic kidney disease, sepsis, total bilirubin, norepinephrine, vasopressin, furosemide, phenylephrine, phosphate, potassium, and platelet count.

**Conclusions:**

Our machine learning-based model effectively identified key covariates of acute kidney injury severity in ICU patients using routinely collected clinical data. The findings provide a foundation for early identification, risk stratification, and targeted intervention strategies for acute kidney injury in critical care settings.

## Introduction

Acute Kidney Injury (AKI) is a rapid decline in kidney function associated with an increase in serum creatinine or a decrease in urine output [1]. Many critically ill patients in intensive care units (ICUs) develop AKI, which contributes to increased morbidity, longer hospital stays, and higher mortality rates [2]. Guidelines classify AKI into three stages based on serum creatinine levels and urine output, with increasing severity correlating with poorer prognosis and higher mortality [3,4]. Given its substantial significance, the identification of AKI and its covariates in ICU settings remains crucial for improving patient outcomes [5].

Current AKI prediction relies on thresholds of serum creatinine and urine output, which fail to capture the dynamic and multifactorial nature of AKI progression [6]. Moreover, clinical scoring systems, including the Sequential Organ Failure Assessment (SOFA) and Simplified Acute Physiology Score II (SAPS II),provide mortality risk stratification but do not specifically assess AKI severity [7]. The pathophysiology of AKI is complex and involves multiple interacting factors, including hemodynamic instability, metabolic imbalances, systemic inflammation, and the presence of comorbid conditions such as cardiovascular disease, chronic kidney disease, and sepsis [8,9]. Additionally, ICU interventions, including mechanical ventilation, vasoactive drug use, and renal replacement therapy, further complicate AKI risk stratification [10,11]. Existing AKI predictive models have several limitations. Many studies use small sample sizes with limited generalizability [12]. Furthermore, current models often incorporate only a limited number of clinical variables, failing to capture a broader spectrum of AKI determinants [13]. The use of machine learning approaches provides an opportunity to enhance predictive accuracy by integrating high-dimensional clinical, laboratory, and comorbidity data [14,15]. Therefore, there is a critical need for integrative, data-driven approaches that leverage high-dimensional clinical data to better characterize AKI severity and improve early risk stratification in ICU patients [15].

The aim of conducting this study was to enhance AKI severity prediction by developing and evaluating a robust framework trained on a large ICU dataset including AKI covariates. By incorporating a comprehensive array of covariates—including vital signs, laboratory parameters, comorbid conditions, and ICU interventions—we sought to develop a clinically applicable model that enhances early AKI risk stratification and informs personalized management strategies. This has the potential to refine existing clinical decision-making tools and improve outcomes for critically ill patients with AKI. Our hypothesis was that integrating demographics, vital signs, lab values, comorbidities, and ICU interventions could be used in AKI severity classification, while providing a more comprehensive representation of AKI complexity.

## Patients and methods

### Study design and data source

We conducted a secondary analysis of cross-sectional data from patients admitted to the ICU. The dataset was selected from the publicly available Medical Information Mart for Intensive Care-IV (MIMIC-IV v3.1) database (https://mimic.mit.edu).The MIMIC-IV contains detailed clinical records of patients admitted to the ICUs of a tertiary Medical Center in Boston, MA, between 2008 and 2022. The dataset, which is fully de-identified, includes extensive patient-level data such as demographic characteristics, vital signs, laboratory parameters, comorbid conditions, therapeutic interventions, and clinical outcomes. The cohort analyzed in our study consisted of adult patients (18 years or older) who were admitted to the ICU for the first time for at least 48 hours between 2017 and 2022.

### Candidate variables

The study dataset included patient identifiers, ICU stay information, and baseline characteristics such as age, gender, race, marital status, and BMI. Clinical data encompass both chronic comorbidities and acute diagnoses, as well as indicators of organ dysfunction derived from diagnostic codes and laboratory criteria. The dataset also included a wide range of laboratory values and vital signs reflecting cardiovascular, respiratory, renal, hepatic, and hematologic function. Additionally, it included medication administration, specifically vasoactive agents and diuretics. This comprehensive dataset was used for characterizing patients with AKI in the ICU setting. Our investigation revealed a lack of complete and reliable data on urinary output for the included patients, limiting our ability to apply urine output criteria for AKI classification. Therefore, we defined AKI severity based on serum creatinine (Cr) concentrations, using established staging criteria. To ensure a comprehensive assessment across the full spectrum of AKI severity, we included patients without AKI (stage 0) alongside those with stages 1–3. Briefly, AKI was defined as either an increase in serum Cr to ≥1.5 times the baseline value or an absolute increase of ≥0.3 mg/dL within 48 hours of ICU stay. AKI severity was then staged as follows: stage 1 included patients with a serum Cr increase to 1.5–1.9 times baseline or an absolute increase of ≥0.3 mg/dL; stage 2 included patients with a serum Cr increase to 2.0–2.9 times baseline; and stage 3 included those with a serum Cr increase to ≥3.0 times baseline, an increase in serum Cr to ≥4.0 mg/dL [16]. Stage 0 AKI patients were identified as those who did not meet any of the serum creatinine-based criteria for AKI and were not coded as having AKI according to the ICD diagnosis codes in the dataset. Baseline serum creatinine was defined as the first available measurement at ICU admission. We included participants with less than 25% missing data in the study variables. Our final dataset included four severity grades, ranging from stage 0 to stage 3, with stage 2 being the smallest subgroup (n = 343) and stage 0 the largest (n = 5116). To avoid a significant imbalance in the response variable (AKI severity), we randomly selected patients so that the largest group contained twice as many patients as the smallest group.

### Ethical considerations

The original raw data was anonymized, preventing any identification of individuals. This study adheres to the principles outlined in ethical protocols. No direct measurements of participant characteristics were conducted, nor were any personally identifiable details collected. Rather, we performed a secondary analysis on de-identified data. Our findings are based on publicly accessible data, and we did not republish any raw data. Ethics approval was obtained from the institutional review board of Shahid Beheshti University of Medical Sciences with the reference number of IR.SBMU.RETECH.REC.1403.896.

### Data analyses

The outcome variable included ordered categories of four severity levels (stages 0–3). We used an ordinal modeling framework that could flexibly accommodate non-linear interactions and high-dimensional predictor spaces. Data preprocessing involved rigorous screening and exclusion of variables based on missingness thresholds, multicollinearity (Spearman’s ρ > 0.8), and class imbalance. After preprocessing, missing data in the retained features were imputed using predictive mean matching, a semi-parametric multiple imputation technique that preserves the empirical distribution and inter-variable relationships. Feature selection was conducted using the Boruta algorithm, a wrapper method around random forest classification that performs all-relevant feature identification through iterative comparison with permuted shadow variables [18]. This method allowed us to identify an initial subset of features with confirmed predictive utility for AKI severity staging. Given the ordinal and multi-class nature of the outcome variable, as well as the non-linear and high-dimensional structure of the predictor space, we selected Extreme Gradient Boosting (XGBoost) as the primary modeling algorithm. Traditional linear models (e.g., logistic regression) were deemed suboptimal due to their limited ability to model complex, non-linear interactions among heterogeneous clinical variables. The XGBoost, a scalable ensemble learning technique based on gradient-boosted decision trees, offers several advantages: it is robust to multicollinearity, can naturally handle missing values, supports non-parametric interactions, and performs intrinsic regularization through shrinkage and column subsampling. Hyperparameter tuning was conducted via 10-fold cross-validation to minimize generalization error and ensure both flexibility and stability of the fitted model. Model performance was evaluated using class-specific precision, recall, and F1-scores, as well as global metrics such as accuracy and mean absolute error on a hold-out test set representing 25% of the data. Model interpretability was facilitated through gain-based feature importance analysis, reflecting the marginal contribution of each predictor to the reduction of the loss function across all boosting rounds. P-values were calculated using the Kruskal-Wallis test or the χ² test, as appropriate. The level of significance was set at the two-tailed α = 0.05. Data analyses and visualization were performed with R software version 4.0.2. for Windows(R Foundation for Statistical Computing, Vienna, Austria. https://www.R-project.org/) and Python version 3.11 and its data analysis libraries(https://pypi.org/).

## Results

### Preprocessing and feature selection

**Fig 1:**
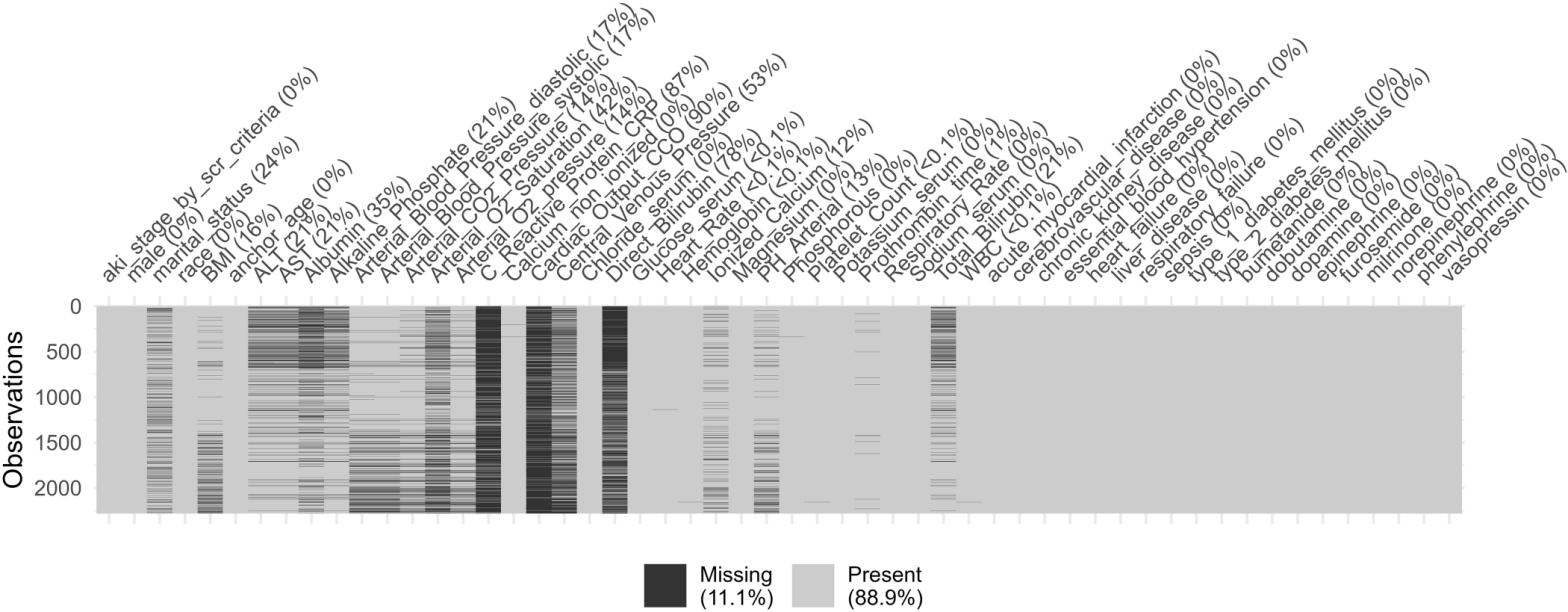
Initial candidate variables and their missing data patterns.

**Fig 2:**
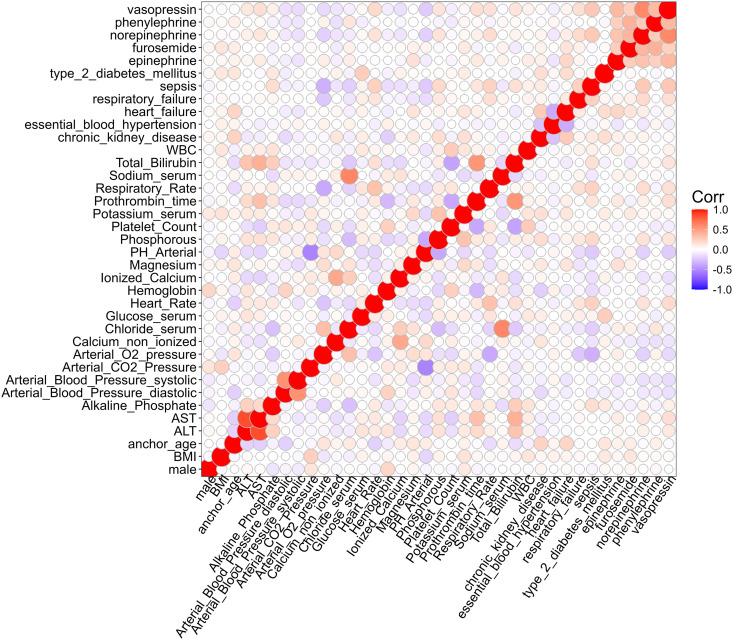
The heatmap of pairwise Spearman’s correlations among study variables. Only the pair ALT-AST showed a correlation coefficient of >0.8.

**Fig 3:**
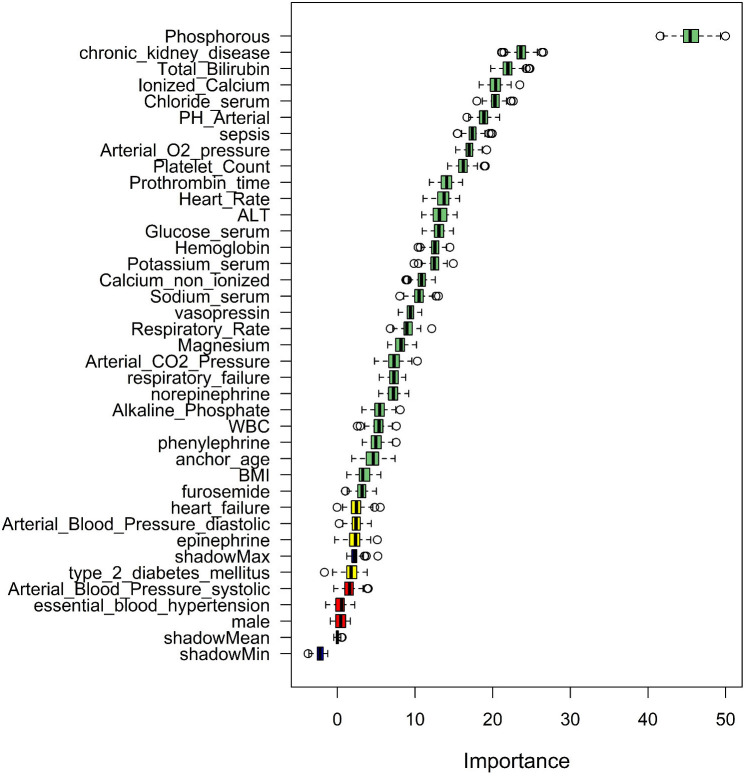
Results of the Boruta feature selection algorithm for AKI severity. The color coding represents the importance of each feature: green indicates features confirmed as important, red represents features confirmed as unimportant, yellow shows features that are tentative, and blue corresponds to the shadow features used for comparison in the Boruta analysis.

There were no duplicate rows in the study dataset. Fig 1 illustrates the initial variables and the patterns of missing data. In total, 11.1% of the initial data were missing. We then excluded variables with >25% missing data from further analysis: cardiac output (89.7% missing), C-reactive protein (87.3%), direct bilirubin (78.0%), central venous pressure (53.0%), arterial O₂ saturation (42.3%), and serum albumin (35.4%). We also decided to exclude marital status, which had a relatively high proportion of missing data (24%) and did not appear to meaningfully influence the outcomes of our analysis. Furthermore, the variable race was excluded due to its high cardinality (30 levels). While tree-based methods are generally robust to class imbalance, variables with low prevalence may contribute little to model performance and interpretability. Therefore, binary features were evaluated for imbalance, and those with fewer than 10% positive cases were excluded from the analysis. Specifically, among comorbidities, liver disease (3.4% positive), type 1 diabetes mellitus (1.8%), cerebrovascular disease (1.1%), and acute myocardial infarction (0.4%) were excluded. Similarly, medication use variables of dobutamine (4.2%), milrinone (3.2%), dopamine (1.7%), and bumetanide (1.3%) were also excluded due to low prevalence. Fig 2 illustrates the heatmap of correlations among the study variables using Spearman’s correlation coefficients. There was a large (ρ = 0.85) and statistically significant correlation between ALT and AST. We excluded AST from further analysis. The remaining dataset had only 4.4% missing values. We imputed missing data using predictive mean matching with a maximum iteration of 5 and used the Boruta algorithm with a confidence level of 0.01 and a maximal runs number of 100 to initially select features associated with AKI stages. Based on the results of the Boruta algorithm (Fig 3), systolic blood pressure, history of essential hypertension, and male sex were excluded from analysis. Table 1 shows the final set of variables entering the study’s multivariable model, and their descriptions for the total sample and AKI subgroups. Univariable comparisons showed significant heterogeneity across AKI stages, indicating potential discriminative power in modeling severity. Variables such as BMI, age, ALT, alkaline phosphatase, glucose, potassium, and total bilirubin demonstrate monotonic trends with increasing AKI severity, suggesting progressive shifts in their distributions as kidney dysfunction advances. In contrast, other variables (e.g., diastolic blood pressure, chloride, and platelet count) show non-monotonic patterns, with intermediate stages sometimes deviating from expected trajectories. The relatively consistent differentiation between stage 0 and stage 3 patients implied strong distributional separation at the extremes. However, overlap is observed between stages 1 and 2 which suggests the classification challenges and clinical and measurement noise inherent in intermediate AKI stages. Categorical variables such as comorbidities and medication usage also show significant variation, with higher prevalence rates in more severe AKI stages, though not always in a linear fashion. Overall, the statistical structure of Table 1 supports stage discrimination at the extremes but reveals blurred group boundaries in the middle ranges of severity. To compensate the complex interrelationships within the dataset, multivariable analysis was conducted using the tree-based XGBoost algorithm.

**Table 1:** Sample description and univariate comparisons of model variables among the study groups.

		AKI stage				
	Total	0	1	2	3	p
n	2281	686	686	343	566	
BMI (kg/m2)	30.4 [26.2,35.2]	29.3 [25.3,33.5]	30.9 [26.9,36.0]	30.5 [25.3,35.0]	31.0 [26.9,36.4]	<0.001*
Age (year)	63.0 [53.0,71.0]	64.0 [55.0,71.0]	65.0 [56.0,73.0]	61.0 [48.0,69.0]	60.0 [50.2,71.0]	<0.001*
ALT (IU/L)	33.0 [17.0,88.0]	24.0 [14.2,49.0]	33.0 [16.2,90.0]	49.0 [22.0,163.0]	44.0 [19.0,147.5]	<0.001*
Alkaline Phosphate (IU/L)	78.0 [57.0,119.0]	69.0 [53.0,99.0]	78.0 [56.0,116.8]	85.0 [57.5,125.5]	89.0 [62.0,140.8]	<0.001*
Diastolic Blood Pressure (mmHg)	58.0 [51.0,68.0]	61.0 [53.0,70.0]	57.0 [50.0,65.0]	61.0 [53.0,71.0]	56.0 [49.0,66.0]	<0.001*
Arterial CO2 (mmHg)	41.0 [36.0,47.0]	41.0 [37.0,46.0]	42.0 [36.0,47.0]	41.0 [35.0,47.0]	40.0 [34.0,48.0]	0.148
Arterial O2 (mmHg)	140.0 [91.0,274.0]	230.0 [121.2,336.0]	149.0 [93.0,282.8]	113.0 [81.5,204.5]	106.0 [81.0,162.2]	<0.001*
Non-ionized Calcium (mg/dL)	8.2 [7.7,8.7]	8.3 [7.9,8.7]	8.2 [7.8,8.7]	8.2 [7.6,8.8]	8.2 [7.6,8.8]	0.015*
Chloride (mEq/L)	104.0 [99.0,108.0]	106.0 [103.0,109.0]	104.0 [100.0,108.0]	103.0 [98.0,108.0]	100.0 [94.0,105.0]	<0.001*
Glucose (mg/dL)	136.0 [111.0,179.0]	127.0 [108.0,150.8]	142.0 [115.0,190.0]	144.0 [113.0,203.0]	140.0 [110.0,190.8]	<0.001*
Heart Rate (bpm)	88.0 [78.0,103.0]	81.0 [73.0,92.0]	88.0 [79.0,104.0]	100.0 [83.5,114.0]	93.0 [80.0,107.0]	<0.001*
Hemoglobin (g/dl)	10.5 [8.6,12.5]	11.3 [9.8,13.0]	10.5 [8.6,12.6]	10.5 [8.6,12.7]	9.4 [7.8,11.5]	<0.001*
Ionized Calcium (mmol/L)	1.1 [1.0,1.2]	1.1 [1.1,1.2]	1.1 [1.1,1.2]	1.1 [1.0,1.2]	1.1 [1.0,1.1]	<0.001*
Magnesium (mg/dL)	2.1 [1.8,2.5]	2.0 [1.8,2.4]	2.2 [1.8,2.6]	2.1 [1.7,2.4]	2.1 [1.9,2.5]	<0.001*
Arterial PH (units)	7.3 [7.3,7.4]	7.4 [7.3,7.4]	7.3 [7.3,7.4]	7.3 [7.3,7.4]	7.3 [7.2,7.4]	<0.001*
Phosphorus (mg/dL)	3.9 [3.1,5.3]	3.3 [2.8,3.9]	4.1 [3.1,5.1]	4.0 [3.1,5.2]	5.8 [4.3,7.3]	<0.001*
Platelet Count (K/uL)	163.0 [111.0,236.0]	170.5 [130.0,227.8]	163.0 [109.0,237.8]	156.0 [94.0,228.0]	155.0 [89.0,241.8]	0.001*
Potassium (mEq/L)	4.4 [3.9,4.9]	4.2 [3.9,4.6]	4.4 [4.0,4.9]	4.4 [3.9,4.9]	4.6 [4.0,5.2]	<0.001*
Prothrombin Time (sec)	15.3 [13.1,18.6]	14.3 [12.5,16.4]	15.8 [13.4,19.1]	15.6 [13.5,20.5]	16.0 [13.3,21.7]	<0.001*
Respiratory Rate (insp/min)	19.0 [16.0,23.0]	17.0 [15.0,20.0]	19.0 [16.0,24.0]	20.0 [16.0,25.0]	20.0 [16.0,25.0]	<0.001*
Sodium (mEq/L)	138.0 [135.0,141.0]	139.0 [136.0,140.8]	138.0 [135.0,141.0]	138.0 [135.0,141.0]	137.0 [132.0,141.0]	<0.001*
Total Bilirubin (mg/dL)	0.7 [0.4,1.6]	0.6 [0.4,0.9]	0.7 [0.5,1.6]	1.0 [0.5,2.3]	0.9 [0.4,4.5]	<0.001*
WBC (K/uL)	12.7 [8.9,17.6]	12.2 [8.9,15.7]	12.7 [8.9,18.1]	12.8 [8.5,18.1]	13.6 [9.3,19.0]	<0.001*
Chronic Kidney Disease	595 (26.1)	48 (7.0)	240 (35.0)	60 (17.5)	247 (43.6)	<0.001*
Heart_Failure	711 (31.2)	150 (21.9)	276 (40.2)	105 (30.6)	180 (31.8)	<0.001*
Respiratory Failure	1213 (53.2)	252 (36.7)	428 (62.4)	203 (59.2)	330 (58.3)	<0.001*
Sepsis	839 (36.8)	78 (11.4)	304 (44.3)	151 (44.0)	306 (54.1)	<0.001*
Type 2 Diabetes Mellitus	712 (31.2)	166 (24.2)	269 (39.2)	90 (26.2)	187 (33.0)	<0.001*
Epinephrine	299 (13.1)	66 (9.6)	122 (17.8)	50 (14.6)	61 (10.8)	<0.001*
Furosemide	1082 (47.4)	331 (48.3)	384 (56.0)	148 (43.1)	219 (38.7)	<0.001*
Norepinephrine	951 (41.7)	155 (22.6)	334 (48.7)	181 (52.8)	281 (49.6)	<0.001*
Phenylephrine	811 (35.6)	275 (40.1)	280 (40.8)	109 (31.8)	147 (26.0)	<0.001*
Vasopressin	548 (24.0)	54 (7.9)	206 (30.0)	120 (35.0)	168 (29.7)	<0.001*

*Significant at p < 0.05.

Values are either presented as median [interquartile range] or count (%).

### The XGBoost model

**Fig 4:**
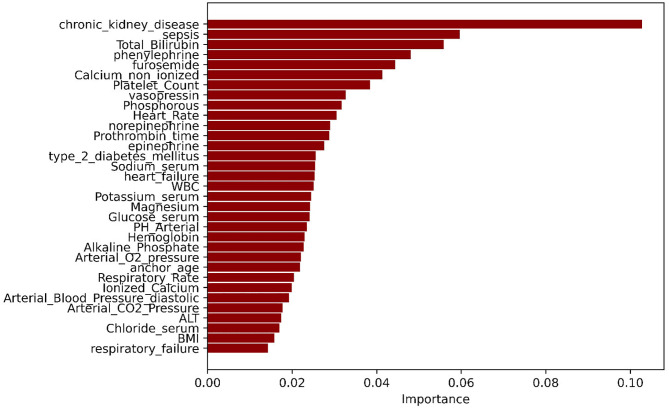
The XGBoost importance plot for the association of the study variables with the severity of AKI.

We developed an XGBoost regression model to predict acute kidney injury (AKI) stages, ranging from 0 (normal) to 3. Hyperparameter tuning was conducted using 10-fold cross-validation, resulting in the optimal parameters: learning rate of 0.01, maximum tree depth of 10, 700 estimators, and a subsample ratio of 0.9. The model achieved a mean absolute error of 0.241. Calibration analysis demonstrated strong agreement between predicted and observed AKI severity levels. The model showed a calibration slope of 0.987 and calibration intercept of 0.065, indicating minimal systematic overestimation or underestimation across the prediction range. In addition, the calibration coefficient of determination was high (R² = 0.821), supporting substantial concordance between predicted and observed AKI stages. Additionally, model performance was evaluated on a test set comprising 25% of the data. Predictions were rounded to the nearest integer stage and compared against the true test labels. Class-specific diagnostic metrics for AKI stages 0–3 are summarized in Table 2. The model showed robust predictive performance on the independent test set, as reflected by a relatively low mean absolute error. Examination of the confusion matrix and class-specific metrics revealed that the model was particularly effective in accurately identifying patients without AKI (stage 0) and those with the most severe form (stage 3), achieving high precision and recall for these categories. In contrast, predictive performance was reduced for stage 2. The model frequently misclassified stage 2 cases as stage 1. While classification accuracy remained relatively strong for stages 0, 1, and 3, this misclassification pattern suggests limitations in the model’s ability to distinguish the clinical profile associated with AKI stage 2. The metrics indicate a reasonably balanced performance across all stages, while the weighted averages reflect the influence of the class distribution in the test dataset. These findings underscore the potential of the model for clinical application in stratifying AKI severity, particularly at the extremes of the disease spectrum. Fig 4 displays a horizontal bar plot showing the feature importance from the XGBoost model developed to predict AKI stages (0–3). Feature importance values are derived from the gain metric, which reflects each feature’s relative contribution to reducing the model’s loss function across all boosting rounds. The importance values are normalized to sum to 1, allowing direct comparison. The most predictive feature is chronic kidney disease, contributing over 10% of the model’s total importance, followed by sepsis and total bilirubin. The plot highlights a long-tail distribution of features with relative importance gradually tapering off.

**Table 2:** Model performance metrics and confusion matrix for the XGBoost model on the test set (N = 594).

Confusion Matrix	Class				Precision	Recall	F1-score
	0	1	2	3			
0 (Normal)	165	14	0	0	0.917	0.922	0.919
1	10	131	38	0	0.682	0.732	0.706
2	5	42	42	0	0.424	0.472	0.447
3	0	5	19	123	1.000	0.837	0.911
Macro Average					0.756	0.741	0.746
Weighted Average					0.793	0.776	0.782
Accuracy		0.776					
Mean Absolute Error		0.241					

## Discussion

This study aimed to advance the understanding of AKI severity among ICU patients by leveraging a high-dimensional integrative approach. We used a large, heterogeneous ICU cohort, integrated a wide spectrum of clinical, biochemical, and therapeutic variables, and used advanced feature selection and modeling techniques tailored for ordinal outcomes.

The selection of variables in this study was guided by both methodological rigor and clinical applicability, prioritizing features that are routinely and reliably available in ICU settings. This approach ensures that the developed model remains practical for real-time implementation without reliance on derived or inconsistently recorded indices. Although composite biomarkers such as the neutrophil-to-lymphocyte ratio may provide additional prognostic value, they were not included due to limitations in data completeness and the study’s emphasis on directly measured, high-availability clinical parameters. Our findings demonstrated that AKI severity is associated with a complex interplay of physiological parameters, comorbid conditions, and therapeutic interventions, rather than any single determinant. The study model highlighted the feasibility and clinical relevance of machine learning-based stratification in the ICU environment. The feature importance ranking underscores several crucial factors that align with the pathophysiology of AKI. Chronic kidney disease, being the top predictor, confirms clinical knowledge that patients with pre-existing kidney damage are highly susceptible to worsening renal function. Sepsis, another leading predictor, reflects the profound impact of systemic infection and inflammatory response on renal perfusion and function, often precipitating AKI. Likewise, elevated total bilirubin may indicate underlying liver dysfunction or multi-organ failure, both associated with poor renal outcomes. The presence of vasoactive medications (e.g., phenylephrine, vasopressin, norepinephrine, epinephrine) and diuretics like furosemide among top features highlights their dual role as both indicators of hemodynamic instability and renal hypoperfusion. The importance of lab values such as calcium, platelet count, phosphorus, and prothrombin time suggests systemic derangements (e.g., electrolyte imbalances, coagulopathy) that often accompany or exacerbate AKI. Demographic and baseline variables like age and BMI ranked lower, indicating that acute physiological and biochemical markers more strongly influence AKI severity. In practice, this model can aid clinicians by prioritizing at-risk patients, especially when integrated into electronic health systems for real-time decision support. The insights also provide a data-driven foundation for targeting modifiable risk factors, improving triage, and tailoring treatment strategies to reduce progression to severe AKI stages. Overall, these findings underscore the value of incorporating granular, multimodal data into AKI severity classification.

Our findings supported the critical role of chronic kidney disease, sepsis, and vasoactive agent exposure as dominant covariates of AKI severity in ICU settings [15,19]. These are compatible with recent epidemiological observations from multicenter studies that emphasize these variables as independent predictors of AKI onset and progression [20,21]. Our model’s identification of total bilirubin and liver dysfunction as key covariates is consistent with nomogram-based work in cirrhotic ICU patients showing hepatic dysfunction as a major predictor of AKI incidence [22]. Similar to our findings, increased potassium and phosphate concentrations have been reported to be associated with advancing AKI stages [23]. Moreover, the impaired ability to separate stages 1 and 2 reflects previously reported classification overlap, which limits discrimination in intermediate AKI stages [24]. This suggests that the current AKI staging criteria may warrant re-evaluation to improve clinical differentiation, although such an investigation lies beyond the scope of our study. The ability of the XGBoost-based model in capturing high-order nonlinearities is consistent with prior machine learning studies that outperformed logistic models in AKI prediction tasks using ICU data [25,26]. The correlation between cardiovascular comorbidities and AKI stages reflects similar associations reported in the critical illness literature, where hemodynamic instability and vasopressor use significantly predicted AKI onset [27]. Our findings suggest that incorporating data-rich, multivariable models into clinical decision support could improve AKI stratification and therapeutic timing in ICU nephrology.

The biological plausibility of the findings is supported by recent literature. A state of diminished nephron reserve and impaired autoregulatory capacity, as seen in chronic kidney disease, amplifies susceptibility to ischemic and nephrotoxic stressors during acute critical illness [28]. Sepsis, a leading contributor to AKI in ICU patients, drives renal injury through microvascular dysfunction, mitochondrial damage, and inflammatory cascades leading to tubular apoptosis and endothelial disruption even in the absence of hypotension [29]. Elevated total bilirubin reflects hepatic-renal interaction and indicates the presence of cholestasis, oxidative stress, or bile acid nephropathy that contributes to tubular epithelial dysfunction and renal oxidative injury [30]. The prominence of vasoactive agents such as norepinephrine and vasopressin suggests hemodynamic perturbations as central drivers of renal hypoperfusion and medullary ischemia, particularly through efferent arteriolar vasoconstriction and redistribution of renal blood flow [31]. Although administered therapeutically, their use reflects underlying circulatory failure, a known precipitant of AKI. Furosemide, while often employed to manage fluid overload, may exacerbate renal hypoperfusion in volume-depleted patients, representing a potential iatrogenic contributor to AKI progression [32].Potassium and phosphorus disturbances are associated with cellular injury and cytokine-mediated ion release, and may exacerbate renal dysfunction by promoting tubular toxicity and systemic complications [33]. Thrombocytopenia likely reflects systemic inflammation and disseminated intravascular coagulation, mechanisms associated with renal capillary rarefaction and cortical ischemia [34]. Prolonged prothrombin time may indicate coagulopathy stemming from hepatic dysfunction or sepsis-associated endothelial derangement, both of which compromise glomerular filtration integrity [35]. Disruption of calcium homeostasis interferes with nephron ion channel function and vasodilatory capacity, contributing to ischemic tubular injury [36,37]. The integration of these covariates provides a pathophysiological and clinical framework for understanding AKI severity in ICU patients.

The present study provided several contributions to AKI research. By modeling AKI severity across all KDIGO stages (0–3) using a high-dimensional set of routinely collected ICU variables, this work improved binary prediction frameworks and captured the progressive nature of renal dysfunction. The integration of advanced feature selection and non-linear modeling enabled identification of clinically relevant covariates within a complex, multifactorial setting. Furthermore, the combined use of multivariable importance measures and stage-wise comparisons provided enhanced interpretability, facilitating a more nuanced understanding of AKI severity in critically ill patients.

### Clinical implications

This study provided clinically applicable data-driven suggestions for stratifying AKI severity in ICU patients using routinely collected electronic health data. By incorporating a multidimensional array of variables—such as comorbidities, vital signs, biochemical markers, and ICU interventions—our model enables earlier risk identification compared to traditional scores. Clinicians should recognize chronic kidney disease, sepsis, elevated bilirubin, and vasopressor use as critical early signals of worsening AKI severity. Importantly, this model enhances differentiation at the extremes of AKI stages, supporting timely intervention in high-risk patients while minimizing overtreatment in mild disease. The approach supports integration of machine learning models into decision support systems [26,38]. Practitioners are suggested to consider incorporating high-dimensional AKI risk models into ICU workflows, especially for early triage, escalation of renal monitoring, and timely nephrology referral.

### Limitations

While our study presents a comprehensive and data-driven approach to stratifying AKI severity in ICU patients, several limitations should be acknowledged. The retrospective nature of the analysis may introduce biases inherent to observational studies, such as unmeasured confounding factors. However, the MIMIC-IV database comprises highly granular data from a large academic hospital over multiple years, enhancing the internal validity of the model. Some potentially informative covariates were excluded due to high missingness, low prevalence, or imbalance, which may have limited the inclusion of certain derived or composite indices. The exclusion of urine output, although a limitation, reduced potential inaccuracies from unreliable charting in real-world ICU settings. This ensured greater consistency and reproducibility using a validated and widely accepted serum creatinine-based framework. A further limitation is that predictor variables were modeled as static without explicit temporal alignment to AKI onset. This limits the model’s ability to capture time-dependent dynamics and constrains its interpretation as a fully prospective predictive tool. While our use of a powerful machine learning algorithm(XGBoost) improves modeling of complex interactions, it may challenge direct interpretability compared to traditional statistical models. To address this, we implemented gain-based feature importance metrics to enhance clinical insight. Further research is warranted to externally validate our findings in an independent large dataset.

## Conclusion

This study presents a model for identifying covariates of AKI severity among ICU patients using a high-dimensional array of routinely available clinical, laboratory, and therapeutic variables. By applying advanced machine learning techniques tailored for ordinal outcomes, we identified key predictors of AKI severity, including chronic kidney disease, sepsis, total bilirubin, electrolyte disturbances, and vasoactive drug administration. Our model demonstrated good discriminative performance, particularly in distinguishing patients at the extremes of AKI severity, thereby supporting its potential for risk stratification in critical care settings. The findings highlight the importance of integrating multifactorial and dynamic data in clinical decision-making. The study provided practical insights for early identification and risk stratification of AKI in the ICU. Future efforts should aim at external validation of such models to enhance AKI surveillance and optimize patient outcomes.
